# Views of medical educators on virtual teaching methods and curriculum changes within an undergraduate psychiatry rotation during the COVID-19 pandemic – a quality improvement project

**DOI:** 10.1192/bjo.2021.606

**Published:** 2021-06-18

**Authors:** Ellen Williams, Nidhi Gupta, Femi Oyebode

**Affiliations:** Birmingham and Solihull Mental Health NHS Foundation Trust

## Abstract

**Aims:**

Medical students at the University of Birmingham have historically undertaken a 9 week psychiatry rotation consisting of clinical placements accompanied by lectures and small group teaching. During the COVID-19 pandemic teaching has been offered in a virtual format and clinical placements have been restricted. Our aim was to survey medical educators regarding these changes, seeking their views on resources and skills required for virtual teaching, student engagement, and adaptations to placements.

**Method:**

73 medical educators who had undertaken teaching or clinical supervision were invited to complete an anonymous online survey during December 2020. The survey consisted of Likert scale and open space questions surrounding changes necessitated by the pandemic. Following survey closure quantitative data were analysed using Google Forms and Excel. Qualitative data from the survey were reviewed by all team members to identify relevant themes.

**Result:**

Overall response rate was 40% (29), with participants from 5 NHS trusts. 72% (21) of educators felt they had adequate equipment and resources to facilitate virtual teaching. 55% (16) felt they had adequate training and skills to use virtual teaching platforms effectively. However only 17% (5) felt that students were able to engage in virtual teaching to the same extent as face to face sessions, and just 35% (10) of educators reported enjoying virtual teaching. 76% (22) agreed that information from the University about adjustments to clinical placements was adequate. 66% (19) felt that there had been adequate support to ensure the safety of students, supervisors and patients. However only 20% (6) felt that students had adequate patient contact and 69% (20) did not feel that students had been able meet their clinical competencies.

**Conclusion:**

Our results suggest that the majority of educators have not enjoyed teaching virtually, and feel that students were less engaged. However educators were able to identify some benefits, such as inviting speakers from outside the local area, improved access to manager's hearings and tribunals and the use of simulated patients. There were also innovative suggestions, such as interactive quizzes and feedback polls within sessions. Most educators felt students had not received adequate patient contact during the pandemic and suggestions for improvement were less readily identified, they included changes to work place based assessments and timetabling. We hope using these results to work with the University to develop resources to support educators using virtual teaching methods, and to consider adjustments to clinical placement while the pandemic persists.

